# A Quality Assessment Method Based on Common Distributed Targets for GF-3 Polarimetric SAR Data

**DOI:** 10.3390/s18030807

**Published:** 2018-03-07

**Authors:** Sha Jiang, Xiaolan Qiu, Bing Han, Wenlong Hu

**Affiliations:** 1 Key Laboratory of Technology in Geo-spatial Information Processing and Application System, Institute of Electronics, Chinese Academy of Sciences, Beijing 100190, China; jiangsha15@mails.ucas.ac.cn (S.J.); han_bing@mail.ie.ac.cn (B.H.); wlhu@mail.ie.ac.cn (W.H.); 2 University of Chinese Academy of Sciences, Beijing 100190, China

**Keywords:** GF-3 satellite, PolSAR, quality assessment, crosstalk, channel imbalance

## Abstract

The GaoFen-3 (GF-3) satellite, launched on 10 August 2016, is the first C-band polarimetric synthetic aperture radar (PolSAR) satellite in China. The PolSAR system of GF-3 can collect a significant wealth of information for geophysical research and applications. Being used for related applications, GF-3 PolSAR images must be of good quality. It is necessary to evaluate the quality of polarimetric data and achieve the normalized quality monitoring during 8-year designed life of GF-3. In this study, a new quality assessment method of PolSAR data based on common distributed targets is proposed, and the performance of the method is analyzed by simulations and GF-3 experiments. We evaluate the quality of GF-3 PolSAR data by this method. Results suggest that GF-3 antenna is highly isolated, and the quality of calibrated data satisfies the requests of quantitative applications.

## 1. Introduction

PolSAR can obtain multidimensional data that describe polarization scattering information of targets. And it has many important applications in target classification [1,2], target detection [3,4], biomass inversion [5,6] and so on. Many countries have paid attention to the development of PolSAR systems, and have launched a series of space-borne PolSAR systems, such as Radarsat-2, TerraSAR-X, ALOS-2, Sentinel-1 and Cosmo-Skymed. In August 2016, China launched its first PolSAR satellite—GF-3—which works at the C band and has 12 imaging modes with resolution up to 1 m [7,8]. All imaging modes of this satellite are available in either left- or right-looking orientation. The fully polarimetric mode provides data with swaths of at least 20 km and ground range resolutions of about 8 m and 25 m. And, there are more than 40 beams with look angle ranging from 18.8° to 42.8° at every looking orientation. Undoubtedly, with the GF-3 satellite put into operation, it will provide a substantial number of polarimetric data for quantitative applications on sea and ocean monitoring, disaster reduction, water conservancy, and meteorology. The performance of quantitative application depends extremely on the quality of polarimetric imagery, so users of GF-3 polarimetric data are very concerned about data quality. Further, it is necessary to evaluate the quality of polarimetric data and achieve the normalized quality monitoring during 8-year designed life of GF-3. 

In the quality assessment of polarimetric data, crosstalks (CTs) and channel imbalances (CIs) are the most important metrics. In general, the quality assessment and polarimetric calibration are performed together. There are many methods for polarimetric calibration. These methods are divided into point-target methods and distributed-target methods. Point-target methods use three calibrators, such as corner reflectors [9] and polarimetric active radar calibrators (PARCs) [10]. Distributed-target methods utilize particular distributed targets. Van Zyl [11] and Ainsworth [12] exploited the target of strict reflection reciprocity to solve distortion parameters, Quegan [13] and Villa [14] proposed methods utilizing distributed targets that satisfy the reflection symmetry and reciprocal conditions to perform parameter monitoring. And, these distributed-target methods often require one triangle corner reflector (TCR) to estimate the co-polarization (co-pol) CI. Since the calibration methods mentioned above can calculate CTs and CIs, it can also be used for quality assessment. In addition, the method by using one TCR to extract parameters that characterize CTs and CIs is the most commonly used in quality assessment, abbreviated as TCR Method.

The calibration method and the TCR method can quantitatively assess the quality of PolSAR data, but the TCR arrangement is time-consuming and laborious, and the required distributed targets need to strictly satisfy the reflection symmetry and (or) reciprocal conditions. Therefore, these methods are limited by the area and revisit frequency. Methods using common objects are needed to achieve convenient and normalized quality assessment. Until now, there are some methods that qualitatively assess data quality by employing the common targets. One of them utilizes the impact of CTs and CIs on polarimetric decomposition and classification. These effects were discussed in article [15,16,17,18]. Detailed conclusions are as follows: CTs result in the polarimetric entropy decreasing and the volume scattering component enhancing, and decrease the classification accuracy for surface-scattering pixels; CIs impact the alpha parameters of H/alpha/A decomposition and enlarge the Freeman-Durden decomposition error; Polarization distortion increases the classification bias of H/alpha/Wishart classification method. Thus, the scattering mechanism and classification results of targets are analyzed to indirectly see the data quality. For example, in the paper [19], quality of PolSAR data was assessed by observing the correctness of objects’ scattering mechanisms which were obtained by H/alpha/A-Wishart classification. Another method got assessment results by measuring the consistency of C_HVVH_, C_VHVH_ and C_HVHV_ as well as the difference between HH and VV [20]. These methods have no strict requirements on the objects, but the magnitude of CIs and CTs cannot be estimated.

According to the current research status, we can see that calibration methods can give a quantitative evaluation, but have low applicability; and existing methods using common targets can only give qualitative results, so these methods cannot achieve the normalized quality assessment of GF3 PolSAR data. In this study, we propose a quality assessment method of PolSAR data based on common distributed targets. It does not depend on calibrators and particular distributed targets, which can meet the need of normalized quality assessment, and can supply more accurate reference for applications than qualitative assessment method by giving quantitative results of CIs and CTs. 

We firstly propose evaluation methods of CIs and isolation, respectively, and give their requirements for scattering characteristics of targets. Furthermore, based on the scattering characteristics of RadarSat-2 polarimetric data, we summarize types of distributed targets that meet requirements, i.e., the forest for amplitude imbalance evaluation and the non-water natural objects for phase imbalance and isolation evaluation. Then, the whole process of the quality assessment considering both CIs and CTs is presented. The accuracy and anti-noise performance of channel imbalance and isolation evaluation method are evaluated by simulations. And, the effectiveness of the whole assessment method is verified by GF-3 experiments. Finally, we comprehensively assess the quality of GF-3 PolSAR data by using this proposed method. 

The reminder of this study is as follows: Section 2 elaborates the quality assessment method of PolSAR data based on common distributed targets. Section 3 presents the results of simulations to evaluate the performance of this method. Section 4 verifies the effectiveness of the whole assessment method and assesses the quality of GF-3 PolSAR data. Conclusions are drawn in Section 5.

## 2. Quality Assessment Method

The polarimetric distortion problem can be illustrated by the following equation:

(1) $$\left[\begin{array}{cc} M_{HH} & M_{HV}\\ M_{VH} & M_{VV} \end{array}\right]=Ae^{j\Phi }\left[\begin{array}{cc} 1 & \delta_{1}\\ \delta_{2} & f_{r} \end{array}\right]\left[\begin{array}{cc} S_{HH} & S_{HV}\\ S_{VH} & S_{VV} \end{array}\right]\left[\begin{array}{cc} 1 & \delta_{3}\\ \delta_{4} & f_{t} \end{array}\right]$$

where $M_{pq}$ is the measured signal for the polarization pq, p stands for receive polarization, q stands for transmit polarization, A and Φ Φ are absolute amplitude and phase factors of system,$\delta_{x}$ are the system CTs, $f_{t}$ is the transmit CI, $f_{r}$ is the receive CI, and $S_{pq}$ is the target scattering value for the polarization pq.

The TCR Method derives CTs and CIs by utilizing normalized scattering matrix of TCR. According to Equation (1), the measured matrix $M_{triangle}$ of the TCR is expressed as:

(2) $$M_{triangle}\approx Ae^{j\Phi }\left[\begin{array}{cc} 1 & \delta_{3}+f_{t}\delta_{1}\\ \delta_{2}+f_{r}\delta_{4} & f_{r}f_{t} \end{array}\right]$$

In the TCR Method, the larger one of $\left|\delta_{3}+f_{t}\delta_{1}\right|$ and $\left|\delta_{2}+f_{r}\delta_{4}\right|$ is defined as CT in image domain and $f_{r}f_{t}$ stands for the imbalance between HH and VV channel. It should be noted that, in the following, the image-domain isolation refers to the CT in image domain.

As shown in Equations (1) and (2), CIs and CTs are coupled in the measured scattering matrix, and it is very difficult to simultaneously estimate CIs and CTs using distributed targets. Therefore, CIs and isolation evaluation are discussed in Section 2.1 and Section 2.2, respectively. Then, by combining the proposed evaluation method of channel imbalance and isolation, the quality assessment method of PolSAR data based on common distributed targets is summarized in Section 2.3, which is abbreviated as CDT Method.

### 2.1. Channel Imbalance Evaluation

In this section, only CIs are considered. According to Equation (1), the amplitude imbalance of transmit channel ${\left|f_{t}\right|}_{L}$ (dB) and the amplitude imbalance of receive channel ${\left|f_{r}\right|}_{L}$ (dB) can be solved as follows:

(3) $$\{\begin{array}{c} {\left|f_{t}\right|}_{L}=\frac{1}{2}\left(\Delta f_{\alpha }+\Delta f_{\beta }\right)+\frac{1}{2}\left({\left|M_{VV}\right|}_{L}-{\left|M_{HH}\right|}_{L}+{\left|M_{HV}\right|}_{L}-{\left|M_{VH}\right|}_{L}\right)\\ {\left|f_{r}\right|}_{L}=\frac{1}{2}\left(\Delta f_{\alpha }-\Delta f_{\beta }\right)+\frac{1}{2}\left({\left|M_{VV}\right|}_{L}-{\left|M_{HH}\right|}_{L}+{\left|M_{VH}\right|}_{L}-{\left|M_{HV}\right|}_{L}\right) \end{array}$$

where ${\left|•\right|}_{L}=10\times \log10\left(\langle {\left|•\right|}^{2}\rangle \right)$, 〈•〉 stands for ensemble average, |•| means the absolute value,$\Delta f_{\alpha }$ and $\Delta f_{\beta }$ are related to the amplitude characteristic of objects, expressed as Equation (4).

(4) $$\{\begin{array}{c} \Delta f_{\alpha }={\left|S_{HH}\right|}_{L}-{\left|S_{VV}\right|}_{L}\\ \Delta f_{\beta }={\left|S_{VH}\right|}_{L}-{\left|S_{HV}\right|}_{L} \end{array}$$

Similarly, the phase imbalance of transmit channel $\theta_{t}$ and the phase imbalance of receive channel $\theta_{r}$ are obtained:

(5) $$\{\begin{array}{c} \theta_{t}=\frac{1}{2}\left(\Delta \theta_{\alpha }-\Delta \theta_{\beta }\right)+\frac{1}{2}\left(P\left(\langle M_{HV}M_{VH}^{*}\rangle \right)-P\left(\langle M_{HH}M_{VV}^{*}\rangle \right)\right)\\ \theta_{r}=\frac{1}{2}\left(\Delta \theta_{\alpha }+\Delta \theta_{\beta }\right)-\frac{1}{2}\left(P\left(\langle M_{HV}M_{VH}^{*}\rangle \right)+P\left(\langle M_{HH}M_{VV}^{*}\rangle \right)\right) \end{array}$$

where *P* represents the complex phase, (*) indicates the complex conjugate, $\Delta \theta_{\alpha }$ and $\Delta \theta_{\beta }$ are related to the phase characteristics of targets, defined as follows:

(6) $$\{\begin{array}{c} \Delta \theta_{\alpha }=P\left(\langle S_{HH}S_{VV}^{*}\rangle \right)\\ \Delta \theta_{\beta }=P\left(\langle S_{HV}S_{VH}^{*}\rangle \right) \end{array}$$

According to Equation (3), we can find that ${\left|f_{t}\right|}_{L}$ and ${\left|f_{r}\right|}_{L}$ can be calculated by using the measured scattering matrix (M) through Equation (7) if $\Delta f_{\alpha }\approx 0$ and $\Delta f_{\beta }\approx 0$. Similarly, $\theta_{t}$ and $\theta_{r}$ can be calculated by using Equation (8) when $\Delta \theta_{\alpha }\approx 0$ and $\Delta \theta_{\beta }\approx 0$.

(7) $$\{\begin{array}{c} {\left|f_{t}\right|}_{L}=\frac{1}{2}\left({\left|M_{VV}\right|}_{L}-{\left|M_{HH}\right|}_{L}+{\left|M_{HV}\right|}_{L}-{\left|M_{VH}\right|}_{L}\right)\\ {\left|f_{r}\right|}_{L}=\frac{1}{2}\left({\left|M_{VV}\right|}_{L}-{\left|M_{HH}\right|}_{L}+{\left|M_{VH}\right|}_{L}-{\left|M_{HV}\right|}_{L}\right) \end{array}$$

(8) $$\{\begin{array}{c} \theta_{t}=\frac{1}{2}\left(P\left(\langle M_{HV}M_{VH}^{*}\rangle \right)-P\left(\langle M_{HH}M_{VV}^{*}\rangle \right)\right)\\ \theta_{r}=\frac{1}{2}\left(P\left(\langle M_{HV}M_{VH}^{*}\rangle \right)+P\left(\langle M_{HH}M_{VV}^{*}\rangle \right)\right) \end{array}$$

The estimation errors of $f_{t}$ and $f_{r}$ are required to be within 0.3 dB in amplitude and less than 4° in phase for GF-3 quality evaluation. Then, according to Equations (3) and (5)**,** requirements of CIs evaluation for targets can be expressed as Equations (9) and (10):

(9) $$\{\begin{array}{c} \left|\Delta f_{\alpha }\right|<0.3\;\mathrm{dB}\\ \left|\Delta f_{\beta }\right|<0.3\;\mathrm{dB} \end{array}$$

(10) $$\{\begin{array}{c} \left|\Delta \theta_{\alpha }\right|<4^{\circ }\\ \left|\Delta \theta_{\beta }\right|<4^{\circ } \end{array}$$

**Figure 1:**
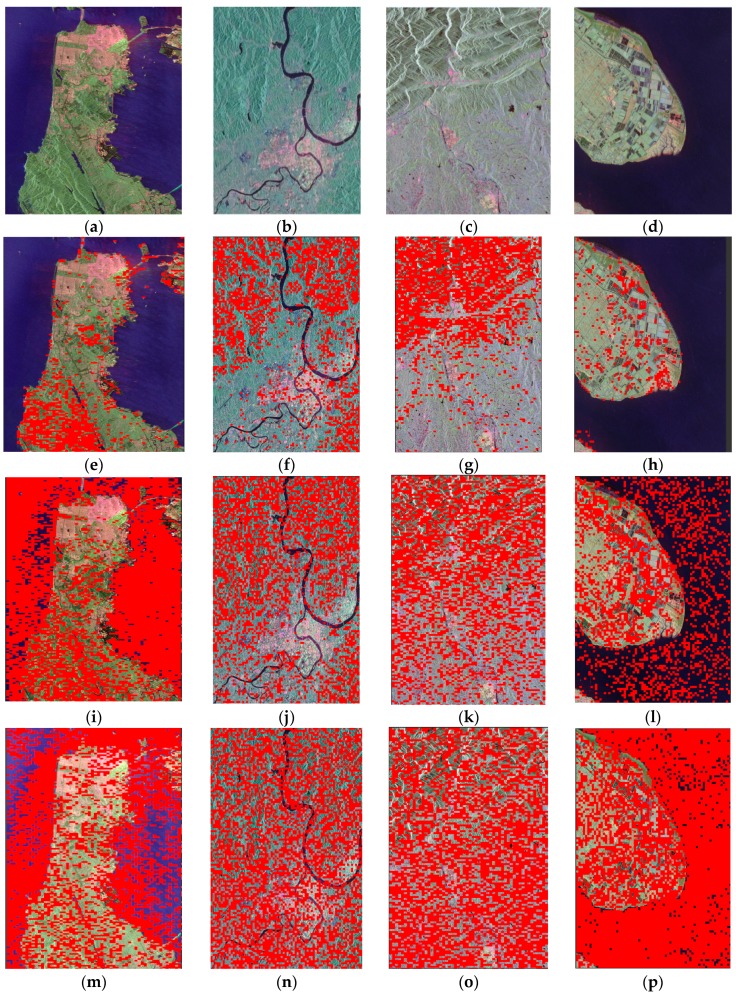
Statistics results of amplitude, phase characteristics and $K_{\rho }$. Areas meeting requirements are marked as red. (**a**–**d**) are the pseudo-color images of data; (**e**–**h**) are amplitude statistics results of (**a**–**d**) respectively; (**i**–**l**) are phase statistics results of (**a**–**d**) respectively, (**m**–**p**) are $K_{\rho }$ statistics results of (**a**–**d**) respectively.

$\left|\Delta f_{\beta }\right|<0.3\;\mathrm{dB}$ and $\left|\Delta \theta_{\beta }\right|<4^{\circ }$ mean the loose reciprocity, which is easy to meet for a monostatic system [14]. Therefore, $\Delta f_{\alpha }$ and $\Delta \theta_{\alpha }$ mainly impact the estimation of CIs. To find the satisfactory distributed targets, 11 calibrated PolSAR images of RadarSat-2 are analyzed. Figure 1e–l show four typical results, areas meeting requirements are marked as red. As shown in Figure 1e–h, It’s obvious that most of areas covered by forest can meet the Equation (9), whereas farmland, bare soil, ocean and urban areas cannot. The experimental result is also confirmed by the reference [21], which points out that most forest or distributed targets possessing the volume scattering mechanism can satisfy the azimuthal symmetry, i.e., $\Delta f_{\alpha }\approx 0$. Therefore, areas covered by forest can be used to estimation of amplitude imbalance ${\left|f_{t}\right|}_{L}$ and ${\left|f_{r}\right|}_{L}$. Next, we discuss the choice of objects for the estimation of phase imbalance. $\langle S_{HH}S_{VV}^{*}\rangle$ of the volume scattering mechanism is about 1/3 in Freeman decomposition model [22], i.e., $\Delta \theta_{\alpha }\approx 0$. Moreover, slightly rough surface such as soil meet $\Delta \theta_{\alpha }\approx 0$ when the incidence angle is less than 60° [23]. These conclusions are consistent with the results that most natural objects (including bare soil, farmland, forest and water) can satisfy the Equation (10) in Figure 1i–l. Nonetheless, the ocean should not be chosen except at vertical incidence, because $\Delta \theta_{\alpha }$ has high value when the look angle becomes large [23]. Consequently, non-water natural objects are selected for estimation of $\theta_{t}$ and $\theta_{r}$. Besides, Figure 1e–l also display that objects used for CIs evaluation are ubiquitous in PolSAR images. 

In summary, areas covered by forest and non-water natural objects are selected to estimate amplitude imbalance and phase imbalance, respectively. Nevertheless, from Figure 1e–l, it can be found that there are some bad pixels in the satisfied area. To prevent these bad pixels to affect CIs estimation, we divide the selected area into blocks with same size and get a group of CIs for each block according to Equations (7) and (8). Then the mode of CIs among all blocks is taken as final estimation of CIs. In that way, it is enough for CIs evaluation that most of selected areas are covered by the satisfied objects. It should be noted that the thinking about the estimation of phase imbalance have been proposed and used on phase calibration [23,24], but we popularize this idea to normalized quality assessment by reducing its requirements for dependent objects.

### 2.2. Isolation Evaluation

In this section, only CTs are considered. In quality assessment, we hope to get a CT value, such as −35 dB, −25 dB, to represent the CT distortion in the imagery instead of the exact value of $\delta_{1}$, $\delta_{2}$, $\delta_{3}$ and $\delta_{4}$. This value can be defined as the equivalent CT and set as $\delta_{v}$ (real number). Then, the relationship between the measured scattering matrix (M) and the real scattering matrix (S) can be expressed as follows:

(11) $$\left[\begin{array}{cc} M_{HH} & M_{HV}\\ M_{VH} & M_{VV} \end{array}\right]=Ae^{j\Phi }\left[\begin{array}{cc} 1 & \delta_{v}\\ \delta_{v} & 1 \end{array}\right]\left[\begin{array}{cc} S_{HH} & S_{HV}\\ S_{VH} & S_{VV} \end{array}\right]\left[\begin{array}{cc} 1 & \delta_{v}\\ \delta_{v} & 1 \end{array}\right]$$

This equation is not the real distortion model but used for impact analysis of equivalent CT on channel correlation. 

The transmit antenna distortion and the receive antenna distortion are assumed to be reciprocal. And, the amplitude of all CTs can be considered as same for equivalent CT evaluation. Then, the real distortion model Equation (1) can be simplified to Equation (12):

(12) $$\left[\begin{array}{cc} M_{HH} & M_{HV}\\ M_{VH} & M_{VV} \end{array}\right]=Ae^{j\Phi }\left[\begin{array}{cc} 1 & \delta_{R}e^{j\theta_{1}}\\ \delta_{R}e^{j\theta_{2}} & 1 \end{array}\right]\left[\begin{array}{cc} S_{HH} & S_{HV}\\ S_{VH} & S_{VV} \end{array}\right]\left[\begin{array}{cc} 1 & \delta_{R}e^{j\theta_{2}}\\ \delta_{R}e^{j\theta_{1}} & 1 \end{array}\right]$$

where $\delta_{R}$ is the amplitude of real CT, $\theta_{1}$ and $\theta_{2}$ are phases of CTs. 

According to the relationship between the measured scattering matrix and real value in the reference [11], it can be reached that CTs cause the variation of correlations between the co-pol and cross-polarization (cross-pol) components. The effects of equivalent CTs and real CTs on the correlation are considered consistent. In that way, we can deduce the Equation (13) of the equivalent CT $\delta_{v}$ with *M* and *S* of natural objects (see Appendix A). The right-side term of the Equation (13) relates only to *M* and *S*:

(13) $$\delta_{v}=\frac{1}{4}\sum_{i=1}^{4}\delta_{v,i}-\frac{1}{4}\sum_{i=1}^{4}\frac{x_{i}}{\gamma_{i}}$$

where:

(14) $$\delta_{v,i}=\frac{\sqrt{{\left|P_{i}\right|}^{2}/A^{4}-y_{i}^{2}}}{\gamma_{i}}$$

(15) $$\{\begin{array}{c} P_{1}=\langle M_{HH}M_{HV}^{*}\rangle \\ P_{2}=\langle M_{HH}M_{VH}^{*}\rangle \\ P_{3}=\langle M_{VV}M_{HV}^{*}\rangle \\ P_{4}=\langle M_{VV}M_{VH}^{*}\rangle \end{array}$$

(16) $$\{\begin{array}{c} \gamma_{1}=\left|<S_{HH}S_{VV}^{*}>\right|+\left|<S_{VH}S_{HV}^{*}>\right|+\langle {\left|S_{HH}\right|}^{2}\rangle +\langle {\left|S_{HV}\right|}^{2}\rangle \\ \gamma_{2}=\left|<S_{HH}S_{VV}^{*}>\right|+\left|<S_{VH}S_{HV}^{*}>\right|+\langle {\left|S_{HH}\right|}^{2}\rangle +\langle {\left|S_{VH}\right|}^{2}\rangle \\ \gamma_{3}=\left|<S_{HH}S_{VV}^{*}>\right|+\left|<S_{VH}S_{HV}^{*}>\right|+\langle {\left|S_{VV}\right|}^{2}\rangle +\langle {\left|S_{HV}\right|}^{2}\rangle \\ \gamma_{4}=\left|<S_{HH}S_{VV}^{*}>\right|+\left|<S_{VH}S_{HV}^{*}>\right|+\langle {\left|S_{VV}\right|}^{2}\rangle +\langle {\left|S_{VH}\right|}^{2}\rangle \end{array}$$

(17) {x1=Re(〈SHHSHV*〉),y1=Im(〈SHHSHV*〉)x2=Re(〈SHHSVH*〉),y2=Im(〈SHHSVH*〉)x3=Re(〈SVVSHV*〉),y3=Im(〈SVVSHV*〉)x4=Re(〈SVVSVH*〉),y4=Im(〈SVVSVH*〉)

Let $K_{\rho }=\frac{1}{4}\sum_{i=1}^{4}\frac{x_{i}}{\gamma_{i}}$, which is related to the real scattering matrix. Equation (13) becomes:

(18) $$\delta_{v}=\frac{1}{4}\sum_{i=1}^{4}\delta_{v,i}-K_{\rho }$$

If $K_{\rho }\approx 0$, then:

(19) $$\delta_{v}=\frac{1}{4}\sum_{i=1}^{4}\delta_{v,i}$$

The numerator of the single addition factor in $K_{\rho }$ is the real of the product of the co-pol component and conjugate cross-pol component, and the denominator is related to the backscattering coefficient of ground objects. Therefore, $K_{\rho }$ actually represents the correlation degree between the co-pol and the cross-pol channel of ground objects. $K_{\rho }\approx 0$ means the low correlation and further indicates that the imag ($y_{i}$) of the correction is small, so the impact of $y_{i}$ on $\delta_{v,i}$ can be ignored. At the same time, real CTs are minimum and $K_{\rho }\approx 0$, so we have the following:

(20) $$\{\begin{array}{c} \langle {\left|M_{HH}\right|}^{2}\rangle \approx A^{2}\langle {\left|S_{HH}\right|}^{2}\rangle \\ \langle {\left|M_{HV}\right|}^{2}\rangle \approx A^{2}\langle {\left|S_{HV}\right|}^{2}\rangle \\ \left|<M_{HH}M_{VV}^{*}>\right|\approx A^{2}\left|<S_{HH}S_{VV}^{*}>\right|\\ \left|<M_{VH}M_{HV}^{*}>\right|\approx A^{2}\left|<S_{VH}S_{HV}^{*}>\right| \end{array}$$

Then, Equation (14) can be rewritten as:

(21) $$\delta_{v,i}\approx \frac{abs\left(P_{i}\right)}{{ϒ}_{i}}$$

where:

(22) $$\{\begin{array}{c} {ϒ}_{1}=\left|<M_{HH}M_{VV}^{*}>\right|+\left|<M_{VH}M_{HV}^{*}>\right|+\langle {\left|M_{HH}\right|}^{2}\rangle +\langle {\left|M_{HV}\right|}^{2}\rangle \\ {ϒ}_{2}=\left|<M_{HH}M_{VV}^{*}>\right|+\left|<M_{VH}M_{HV}^{*}>\right|+\langle {\left|M_{HH}\right|}^{2}\rangle +\langle {\left|M_{VH}\right|}^{2}\rangle \\ {ϒ}_{3}=\left|<M_{HH}M_{VV}^{*}>\right|+\left|<M_{VH}M_{HV}^{*}>\right|+\langle {\left|M_{VV}\right|}^{2}\rangle +\langle {\left|M_{HV}\right|}^{2}\rangle \\ {ϒ}_{4}=\left|<M_{HH}M_{VV}^{*}>\right|+\left|<M_{VH}M_{HV}^{*}>\right|+\langle {\left|M_{VV}\right|}^{2}\rangle +\langle {\left|M_{VH}\right|}^{2}\rangle \end{array}$$

When $K_{\rho }$ of objects is close to zero, the equivalent CT can be calculated by Equations (19) and (21). Considering the error of GF-3 isolation evaluation, the absolute value of $K_{\rho }$ should be lower than 0.005. First, the artificial targets are excluded because the Equation (13) is derived for natural objects. Then, 11 calibrated PolSAR images of RadarSat-2 were analyzed to find satisfied natural objects. Figure 1m–p show four typical results. These results imply that most natural objects except for some ocean meet the requirement. Water body isn’t selected, because the cross-pol water signatures are usually low and the SNR is close to or even below zero [25]. Finally, non-water natural objects are used for the isolation evaluation. Similar to the CIs evaluation, there are some bad pixels in the satisfied area. Therefore, the selected area is divided into blocks. The final estimated isolation is the mode of isolations among all blocks. Specially, according to Equation (11), the CT in image domain measured by TCR Method is $2\delta_{v}$. Therefore, double $\delta_{v}$ estimated by the proposed method is the image-domain isolation and can be compared with the result of TCR Method. 

### 2.3. The Whole Process

**Figure 2:**
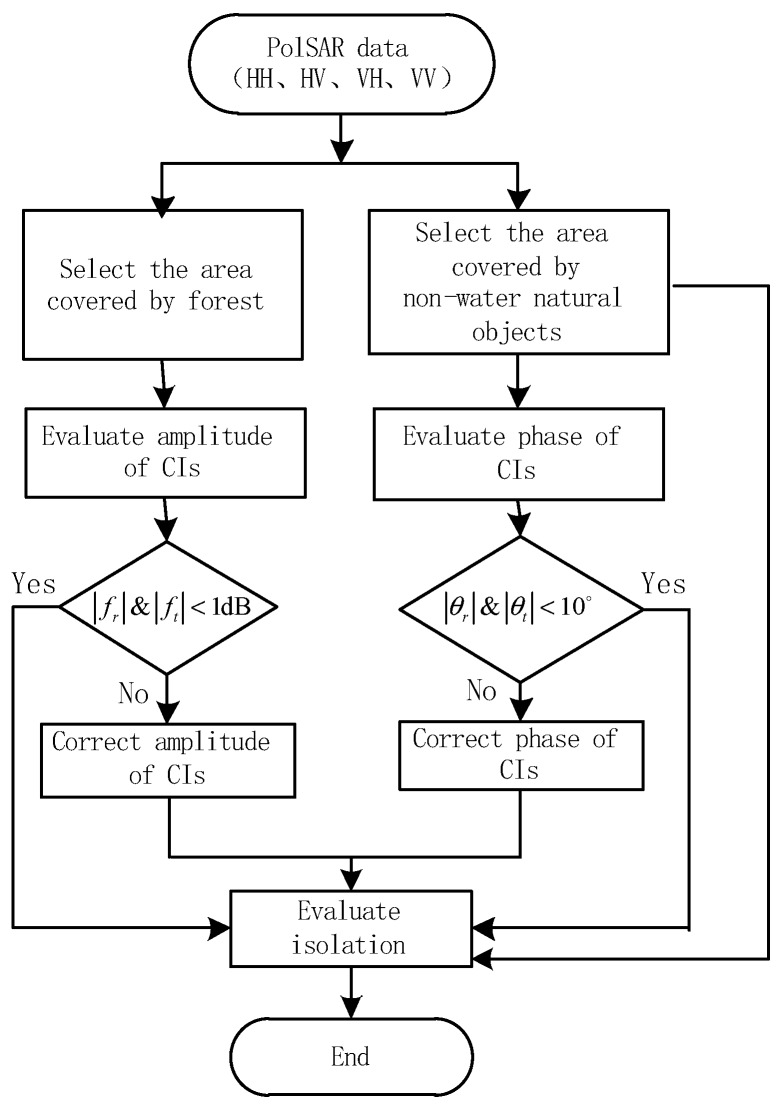
The flowchart of the proposed quality assessment method.

Section 2.1 and Section 2.2 respectively propose evaluation methods of CIs and isolation, but the measured scattering matrix is simultaneously affected by CIs and CTs in actual system. Experimental results (see Section 3.1) demonstrate that the CT of less than −15 dB does not affect the performance of the CI evaluation method. Usually, the CT of an actual system is less than −15 dB [20], so the CIs can be estimated and corrected firstly. Then, the image-domain isolation needs to be evaluated based on the distortion model expressed as:

(23) $$M=Ae^{j\Phi }\left[\begin{array}{cc} 1 & \delta_{1}\\ \delta_{2}/f_{r} & \Delta f_{r} \end{array}\right]\left[\begin{array}{cc} S_{HH} & S_{HV}\\ S_{VH} & S_{VV} \end{array}\right]\left[\begin{array}{cc} 1 & \delta_{3}/f_{t}\\ \delta_{4} & \Delta f_{t} \end{array}\right]$$

where $\Delta f_{r}$ and $\Delta f_{t}$ are residual CIs, which caused by errors of CIs estimation. Simulation results (see Section 3.2) show that $\Delta f_{r}$ and $\Delta f_{t}$ have little impact on the isolation evaluation. CTs of actual system are at the level of negative tens of decibels and amplitudes of $f_{r}$ and $f_{t}$ are less than 2 dB, so the impact of amplitudes of $f_{r}$ and $f_{t}$ on isolation evaluation is negligible. Phases of $f_{r}$ and $f_{t}$ can be classified into the phases of CT. This method can still estimate a valid isolation in the presence of phase of CT (see Section 3.1 and Appendix B). Consequently, after the CI correction, the isolation can be rightly estimated by the proposed method. The whole procedure of the quality assessment consists of two steps: (1) Evaluation and correction of CIs by the method proposed in Section 2.1; (2) Evaluation of isolation by the method proposed in Section 2.2. The specific process is shown in Figure 2. 

## 3. Analysis and Verification by Simulations

**Figure 3:**
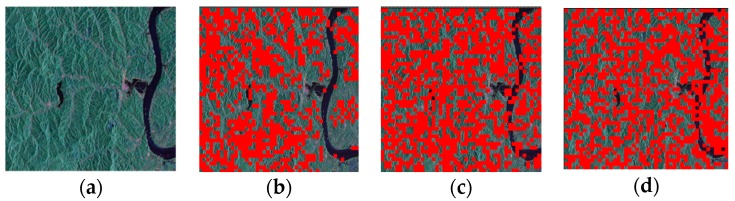
Calibrated RadarSat-2 PolSAR data selected as the test site. These images are (**a**) the pseudo-color image; (**b**) amplitude characteristics; (**c**) phase characteristics; and (**d**) results of $K_{\rho }$. Here, areas meeting requirements are marked as red.

In this section, simulations are carried out to verify the effectiveness of the CI and isolation evaluation methods, respectively. Further, influences of CIs and isolation on each other’s estimation are also analyzed for the proposal of the complete quality assessment procedure. RadarSat-2 PolSAR products the data with globally recognized high quality and works at C-band same with GF-3 satellite [26], so the calibrated RadarSat-2 product is treated as the truth data. In Section 3.1 and Section 3.2, we impose manual CTs and CIs to simulate the distorted data. The selected data, which is shown in Figure 3, was observed for Jiangxi, China, on March 2016. In experiments of this section, when images need to be divided into blocks, the size of all blocks is same (namely, 100×100 pixels). To simplify the analysis, $f_{r}=f_{t}=f$ is assumed in simulations.

### 3.1. Effectiveness Verification under Different Cases

#### 3.1.1. Ideal Case

In the ideal case (noise-free), the effectiveness of the channel imbalance and isolation evaluation methods is respectively verified. 

• CIs Evaluation

**Figure 4:**
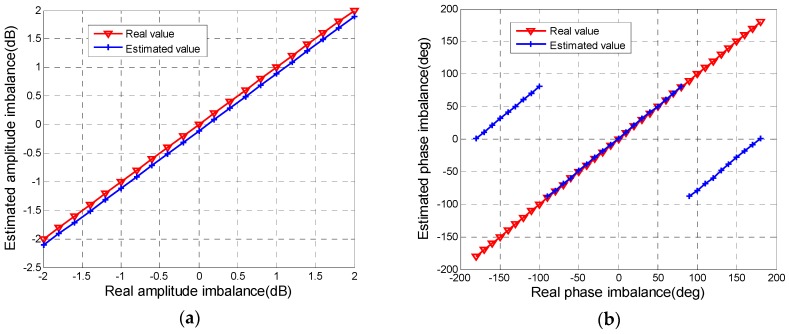
Relationships between the estimated CI and the real value: (**a**) amplitude of f; (**b**) phase of f.

We impose f with the linear amplitude from −2 dB to 2 dB and the linear phase from –*π* to π. Relationships between the amplitude and phase of f estimated by the proposed method and the real values are shown in Figure 4. In Figure 4a, the distance between the estimated line and the truth line is about 0.1 dB, which means that the selected area is very satisfied with Equation (9). That is, the estimation error of amplitude imbalance really can be controlled within 0.3 dB by selecting forest area. In Figure 4b, when the absolute value of phase of f is over π/2, there is a difference of about 180° between the estimated phase and the true value due to the phase ambiguity. The effect of the 180° error in practical situations is minimal, as it merely causes the component of radiation linearly polarized at 45° to be interpreted as being polarized at 135°, and the reverse [24]. So we temporarily ignore this error for quality assessment. In that way, the estimated line and the truth line basically coincide with the real phase of f ranging from −π to π. Hence, evaluated results of phase imbalance can reflect the real phase distortion by selecting non-water natural objects.

• Isolation evaluation

**Figure 5:**
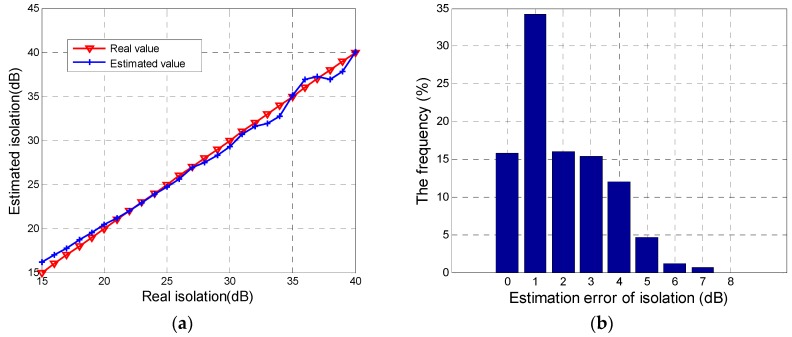
Results of estimated isolation: (**a**) the relationship between the estimated value and the real value with zero-phase CTs; (**b**) the histogram of estimation errors with CTs level of −20 dB and non-zero phase.

First, we impose $\delta_{R}$ with the linear value from −40 dB to −15 dB and $\theta_{1}=\theta_{2}=0$ based on Equation (12). Under the impact of zero-phase CTs, the isolation estimated by the proposed method and the real value are shown in Figure 5a. It suggests that, though the isolation changes from 15 dB to 40 dB, the difference between the estimated isolation and the real value keeps excellently within 1 dB, which proves the correctness and feasibility of the proposed method in Section 2.2. Then, the accuracy of the isolation evaluation method is evaluated by simulating real CT distortion, where $\delta_{R}$ is set as −20 dB and both $\theta_{1}$ and $\theta_{2}$ independently vary from −π to π. A total of 1369 combinations of $\theta_{1}$ and $\theta_{2}$ are simulated. Estimation errors of image-domain isolation from 1369 simulations are calculated and the histogram of these errors is shown in Figure 5b. It can be seen that all errors do not exceed 7 dB and probability of 1 dB error is the highest. Results of error within 5 dB are more than 98%. Therefore, when the phase of CTs isn’t zero, there is an estimation error within 5 dB in most cases. The error of lower than 5 dB can be tolerated by GF-3 isolation evaluation. That is, the proposed method can get an image-domain isolation correctly representing CT distortion in the actual data, which is consistent with the derivation in Appendix B. 

#### 3.1.2. With Noise

**Figure 6:**
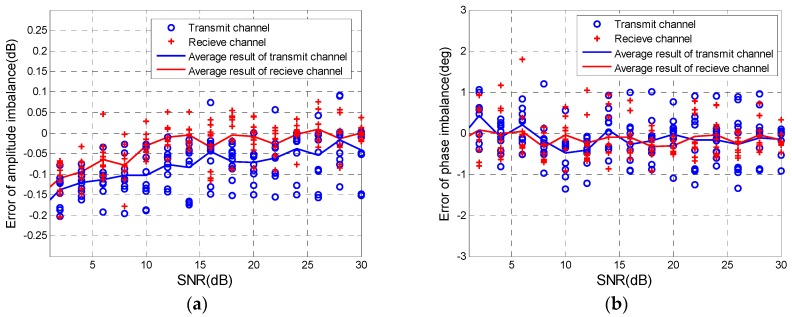

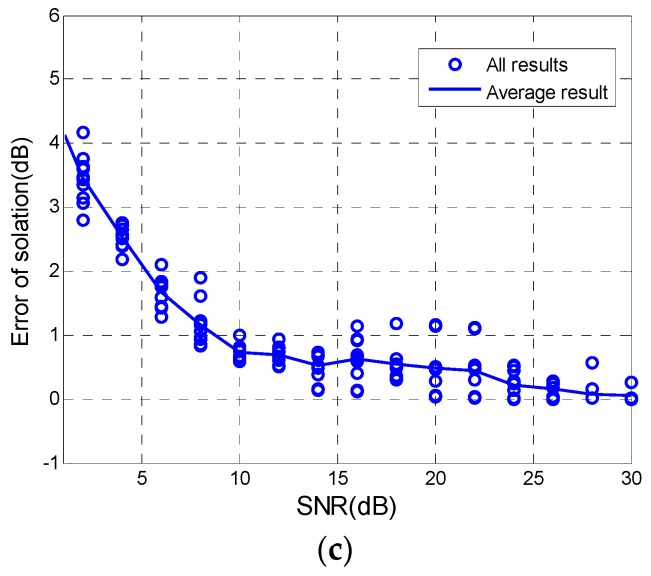
Variations of relative errors of (**a**) amplitude imbalance; (**b**) phase imbalance and (**c**) isolation with the SNR.

The actual PolSAR system is often affected by noise. The anti-noise performance of the proposed method needs to be analyzed. The additive Gaussian noise with the linear SNR from 1 dB to 30 dB was added into the simulated distortion data where the amplitude of f is 1.5 dB, the phase of f is 20°, and CT is −25 dB and zero-phase. And 10 random experiments are conducted. Relative errors between the estimated values with noise and noise-free estimation are shown in Figure 6. As for the amplitude imbalances evaluation in Figure 6a, the variation of relative error in terms of SNR is obvious. Moreover, the tendencies of transmit channel and receive channel are coincident. When the SNR is smaller than 10 dB, amplitude imbalances of transmit channel and receive channel deviate the noise-free estimate values over 0.05 dB and 0.1 dB. In Figure 6b, relative errors of phase imbalances fluctuate in the vicinity of zero and are mostly within 1°, which indicates that noise has no impact on the phase imbalances evaluation. Figure 6c displays that the relative error of isolation is greater as the SNR decreasing, but the error is less than 1 dB when the SNR is more than 10 dB. Combining results of Figure 6a–c, it can be summarized that the noise does not basically impact the evaluation of CIs and isolation when the SNR is more than 10 dB. The SNR of forest and grassland selected by CDT Method is generally greater than 10 dB [20], so this method has good performance in presence of noise. 

### 3.2. Impact Analysis of Isolation and CIs on Each Other’s Evaluation

In Section 3.1, the effectiveness of the channel imbalance and isolation evaluation methods is respectively verified. However, quality assessment needs to simultaneously obtain values of CI and isolation, so influences of isolation and CIs on each other’s evaluation are analyzed in order to support conclusions in Section 2.3.

**Figure 7:**
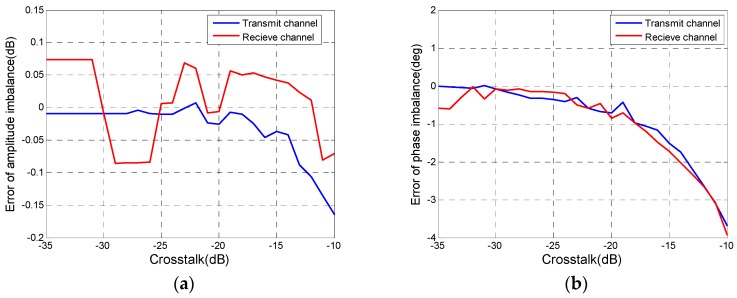
Variations of relative error of (**a**) amplitude imbalance and (**b**) phase imbalance with the CT.

Firstly, the influence of CTs on the CIs evaluation is analyzed. Without considering the phase of CTs, $\delta_{R}$ with the linear amplitude from −35 dB to −10 dB was put into the simulated distorted data where f is 1.5 dB in amplitude and 20° in phase. The relative error between estimated CIs with the CT and these with zero CT are shown in Figure 7. As expected, CTs causes all lines in Figure 7 to show a downward trend, which means that the larger the CT and the greater the impact on the CIs evaluation. However, when CT is less than −15 dB, the relative error of CI is always within ±0.1 dB and ±2°, which is sustainable for quality assessment. In general, CTs of the antenna don’t exceed −15 dB. Hence, results of this simulation support the conclusion in Section 2.3 that the evaluation method of CIs can be used in PolSAR data with CTs. 

**Figure 8:**
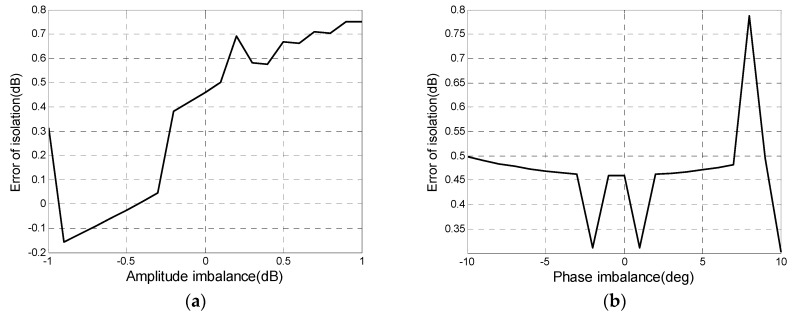
Impacts of (**a**) amplitude imbalance and (**b**) phase imbalance on isolation evaluation.

Next, the impact of CIs on isolation evaluation is analyzed. In the process of the whole quality assessment, which is shown in Figure 2, CIs are estimated and corrected before isolation evaluation, which leads to residual CIs with amplitude below 1 dB and phase within 10° remained in polarimetric images. The CT is a small value, so it is necessary to analyze the influence of residual CIs on the isolation evaluation. CIs with the linear amplitude from −1 dB to 1 dB and linear phase from −10° to 10° were added into distortion data with an isolation level of 20 dB. Relative errors between estimated isolation with CIs and those without CIs are shown in Figure 8. Figure 8a,b reveal that the absolute value of errors keeps less than 1 dB. We can think that the residual CIs don’t affect the isolation estimation, i.e., $\Delta f_{r}$ and $\Delta f_{t}$ in Equation (23) are considered as zero.

## 4. Verification and Quality Assessment of GF-3 PolSAR Data

In this section, two experiments of GF-3 PolSAR data are conducted. Firstly, the CDT method and the TCR method are applied to the un-calibrated data and the calibrated data. All of these data contain artificial TCRs. Distributed-target methods make some hypothesis about distributed targets, so the point-target methods are considered to be more accurate [11]. Hence, the result of the TCR method is taken as true value and compared with the value estimated by the CDT method to further investigate the effectiveness of the whole assessment method. Secondly, the CDT method is used on calibrated PolSAR data of GF-3 satellite to comprehensively assess the current quality of GF-3 PolSAR data. Noted, in this section, the estimate of isolation refers to the CT in image domain.

### 4.1. Verification through Comparing with TCRs

The CDT method and TCR method are applied to four groups of data with TCRs observed for Inner Mongolia, China. Information and quality assessment results of these data are shown in Table 1 and Table 2, respectively. In Table 2 where “Mode” means different beam and “Diff” means the difference of results between the CDT method and TCR method, there are two scenes of uncalibrated data (No. 1 and No. 2) and two scenes of calibrated data (No. 3 and No. 4). 

As shown in Table 2, by comparing the quality assessment results of CDT Method with the results of the TCR method, it is summarized that differences of $\left|f_{t}f_{r}\right|$ and $\theta_{t}+\theta_{r}$ are less than 0.2 dB and within 8°, and the deviation of the isolation is within 4 dB, which can meet the requirement of quality assessment of GF-3 PolSAR data. These results imply that the proposed method is effective for real data. Besides, $\theta_{t}+\theta_{r}$ of No. 2 data is 0.03° estimated by TCR method, which implies that the phase error is very low. In contrast, results of the CDT method show that $\theta_{t}$ and $\theta_{r}$ are at the level of tens of degrees. Therefore, the evaluations of phase imbalances by using the TCR method are insufficient when $\theta_{t}$ and $\theta_{r}$ are similar in absolute value but opposite in sign. This problem also exists in the evaluation of amplitude imbalance. Compared with the TCR method, the CDT method has the advantage of obtaining the transmit CIs and receive CIs simultaneously.

### 4.2. Quality Assessment

According to results in Table 2, the comparison between the CDT method and the TCR method further validates the effectiveness of the proposed method. On the other hand, we can have a sketchy knowledge of the quality of GF-3 polarimetric data. So, from the angle of quality assessments, we analyze the results of Table 2. Results of No. 1 and No. 2 data show that both amplitude and phase of CI are not same under different look angle, as might be expected. It makes no sense to discuss the isolation gap of them because the CT is too small (<−35 dB) [14]. After comparing the polarimetric quality after and before calibration, three points are got as follows: (1) the high isolation is kept; (2) the relatively low amplitude imbalance is maintained within 0.5 dB, even less than 0.1 dB; (3) significant phase imbalance in un-calibrated data has been greatly decreased to lower than 2.2°. In additions, isolation of No. 1 and No. 2 data is already high before polarimetric calibration. To investigate the isolation of other beams, the CDT Method is applied to 20 uncalibrated images with look angle ranging from 21° to 42.8°. The isolations of these un-calibrated data are similar with the results of calibrated data in the next analysis and are not displayed. All isolations of un-calibrated data are higher than 36 dB, so the GF-3 antenna is highly isolated. A diagonal distortion matrix (with zero CTs) can be used for a convenient but still accurate calibration [27]. 

**Figure 9:**
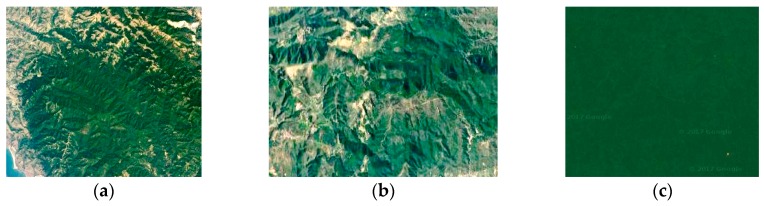
Optical imageries of selected areas of data in Table 3. (**a**–**c**) respectively match the data of No. 1–3 in Table 3.

**Figure 10:**
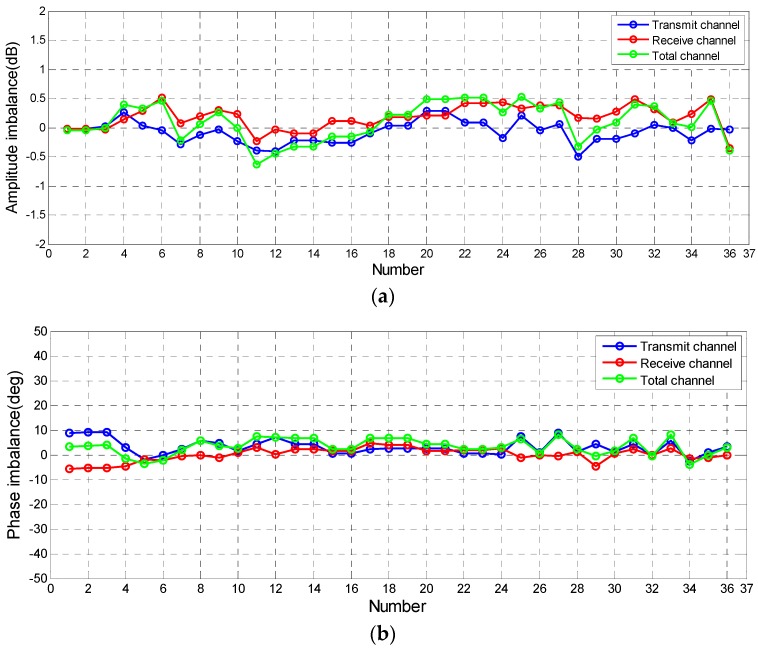

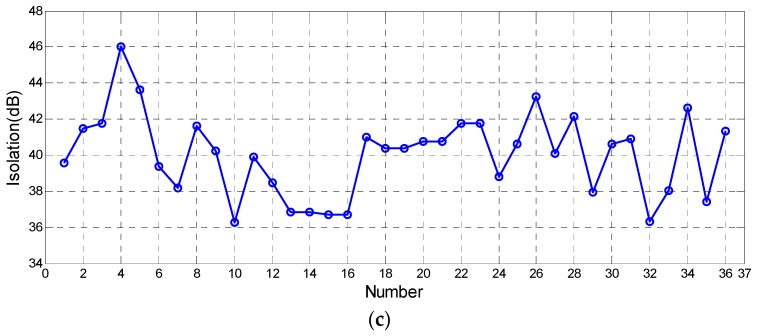
Results of quality assessment of calibrated data: (**a**) the amplitude imbalance; (**b**) the phase imbalance; and (**c**) isolation.

The quality assessment of calibrated data with TCRs in Table 2 reveals the preliminary conclusion that calibrated data have good quality. To fully confirm this conclusion, more different PolSAR data need to be assessed. The CDT method can be applied to common images because it doesn’t depend on TCRs and particular distributed targets. Quality of 36 calibrated PolSAR images is analyzed by using this method to more comprehensively assess the polarimetric data quality of GF-3 satellite. These polarimetric data are mainly covered by natural objects and 6 groups of them were observed for rainforest. These data cover 16 beams, which almost uniformly distribute from low to high look angle. Moreover, they cover multiple bandwidths and pulse widths, and span time of six months. Therefore, the quality assessment of these data can represent the GF-3 polarization system. Table 3 gives the information of three groups of experimental data. Figure 9 shows the optical imagery of data in Table 3. Assessment results of these data are shown in Figure 10. 

In Figure 10, imbalances of transmit channel and receive channel refer to the $f_{t}$ and $f_{r}$. The imbalance of total channel denotes the difference between VV and HH channel. In Figure 10a, although there are some differences between different data, all values of amplitude imbalances are basically maintained within 0.5 dB. Table 2 suggests that the receive channel and the transmit channel have non-negligible relative phase error with the level of tens of degrees. From Figure 10b, it can be seen that, despite the look angle or bandwidth of data are different, the phase imbalances of all data observed at different time and orbits do not exceed 10°, including transmit channel and receive channel, which have been decreased by the polarimetric calibration. As for isolation of calibrated data, Figure 10c implies that the isolations of all data are high (over than 36 dB), which is consistent with the result before calibration. In summary, the results of quality assessments by using this proposed method are: isolation greater than 36 dB, channel imbalance of within 0.5 dB in amplitude and within 10° in phase. These results meet the original quality requirements of GF-3 PolSAR data. 

We may obtain the following conclusions about the quality of GF-3 PolSAR data:

1. Before polarimetric calibration, isolation of data is always less than 36 dB, which indicates that the GF-3 antenna is highly isolated.
2. The channel imbalances are not same under different look angle, and there are non-negligible phases of CIs in the un-calibrated PolSAR data.
3. After polarimetric calibration, the phase imbalances are significantly decreased. The amplitude and phase of the CI are basically maintained within 0.5 dB and 10°, and the isolation is higher than 36 dB, which meet the expected requirement of GF-3 polarimetric performance.

## 5. Conclusions

In this study, a quality assessment method based on common distributed targets for GF-3 polarimetric SAR data is proposed. The effectiveness and anti-noise ability of the method are demonstrated by simulations and GF-3 experiments. These experiments show that the method does have the performances that the estimation error of imbalances between H and V channel is less than 0.3 dB in amplitude and less than 4° in phase, and the isolation error is within 4 dB. These accuracies are sufficient for quality assessment. This method uses common natural objects such as forest and grassland instead of calibrators and particular distributed targets, and quantitatively assesses PolSAR data quality. It finally achieves the purpose of convenient and normalized quality assessment, and provides a means for long-term monitoring and evaluating the quality of a large amount of PolSAR data for GF-3 satellite.

Quality of GF-3 PolSAR data is assessed by this method. Assessment results suggest that the GF-3 antenna is highly isolated, higher than 36 dB, and the amplitude and phase of channel imbalances are basically maintained within 0.5 dB and 10°. Therefore, at present, the quality of GF-3 satellite in CIs and CTs meets the expected requirements for quantitative applications. Besides, this method is already used in “GF-3 ground processing system” to normally monitor the data quality.

## Figures and Tables

**Table 1:** Information of GF-3 PolSAR data with TCRs.

No.	Date	Look Angle (deg)	Direction	Bandwidth (MHz)	Size (Pixel)	Resolution (m)	Location
1	07 September 2016	36.41	ASC	40	6028 × 6561	8 × 8	Inner Mongolia, China
2	07 September 2016	29.36	DEC	60	7919 × 7501	8 × 8	
3	07 November 2017	35.50	ASC	40	4708 × 6165	8 × 8	
4	15 August 2017	41.18	ASC	30	5977 × 3775	8 × 8	

**Table 2:** Quality assessment results of data with TCRs by using the CDT method and TCR method.

No.	Mode	Calibration	Method	$\left|f_{t}\right|$ (dB)	$\theta_{t}$ (deg)	$\left|f_{r}\right|$ (dB)	$\theta_{r}$ (deg)	$\left|f_{t}f_{r}\right|$ (dB)	$\theta_{t}+\theta_{r}$ (deg)	Isolation (dB)
1	Q17	NO	CDT	0.38	90.2	0.16	−67.4	0.54	22.8	38.8
			TCR					0.48	30.5	39.3
			Diff					0.06	7.7	0.5
2	Q9	NO	CDT	−0.18	−79.3	0.08	76.2	−0.1	−3.1	42.3
			TCR					0.03	−0.3	38.9
			Diff					0.13	−2.8	3.4
3	Q15	YES	CDT	−0.19	4.2	0.16	−4.6	−0.04	−0.4	37.9
			TCR					0.04	2.1	37.1
			Diff					−0.08	−2.5	0.8
4	Q25	YES	CDT	0.3	−1.2	−0.34	−4.2	−0.04	−5.4	40.7
			TCR					−0.05	1.7	39.9
			Diff					0.01	7.1	0.8

**Table 3:** Basic parameters of data after polarimetric calibration.

Number	Mode	Look Angle (deg)	Bandwidth (MHz)	Direction	Description
1	Q1	18.90	60	ASC	American
2	Q11	31.70	40	ASC	Italy
3	Q25	41.18	30	DEC	Rainforest

## Appendix A

For natural objects, the detailed derivation about Equation (13) is given.

According to Equation (11) and ignoring the item of $\delta_{v}^{2}$, we get the correlation between $M_{HH}$ and $M_{HV}$:

(A1) $$\langle M_{HH}M_{HV}^{*}\rangle =A^{2}\left(<S_{HH}S_{VV}^{*}>+\delta_{v}\langle {\left|S_{HH}\right|}^{2}\rangle +\delta_{v}<S_{HH}S_{VV}^{*}>+\delta_{v}<S_{VH}S_{HV}^{*}>+\delta_{v}\langle {\left|S_{HV}\right|}^{2}\rangle \right)$$

Due to the results, which are shown in Figure 1i–l, that the phases of $\langle S_{HH}S_{VV}^{*}\rangle$ and $\langle S_{HV}S_{VH}^{*}\rangle$ of natural objects are about zero, Equation (A1) can be simplified to the following line:

(A2) $$\langle M_{HH}M_{HV}^{*}\rangle =A^{2}\left(<S_{HH}S_{HV}^{*}>+\delta_{v}\left[\langle {\left|S_{HH}\right|}^{2}\rangle +\left|<S_{HH}S_{VV}^{*}>\right|+\left|<S_{VH}S_{HV}^{*}>\right|+\langle {\left|S_{HV}\right|}^{2}\rangle \right]\right)$$

Then, the Equation (A3) is obtained by calculating the energy of $\langle M_{HH}M_{HV}^{*}\rangle$:

(A3) $${\left|P_{1}\right|}^{2}=A^{4}\left[{\left(x_{1}+\delta_{v}\gamma_{1}\right)}^{2}{+y}_{1}^{2}\right]$$

where $P_{1}$, $x_{1}$, $y_{1}$ and $\gamma_{1}$ have the same meaning as in Equations (15)–(17).

Further, the solution Equation (A4) is obtained:

(A4) $$\delta_{v}=\frac{\sqrt{{\left|P_{1}\right|}^{2}/A^{4}-y_{1}^{2}}}{\gamma_{1}}-\frac{x_{1}}{\gamma_{1}}$$

Using the expression in Equation (14), Equation (A4) can be abbreviated as:

(A5) $$\delta_{v}=\delta_{v,1}-\frac{x_{1}}{\gamma_{1}}$$

Similarly, according to the correlations of $M_{HH}$ and $M_{HV}$, $M_{VV}$ and $M_{VH}$, $M_{VV}$ and $M_{HV}$, the following three equations are obtained:

(A6) $$\{\begin{array}{c} \delta_{v}=\delta_{v,2}-\frac{x_{2}}{\gamma_{2}}\\ \delta_{v}=\delta_{v,3}-\frac{x_{3}}{\gamma_{3}}\\ \delta_{v}=\delta_{v,4}-\frac{x_{4}}{\gamma_{4}} \end{array}$$

where all the parameters have the same meaning as in Equations (14)–(17).

Equation (A7) is obtained by combing Equation (A5) with Equation (A6).

(A7) $$\delta_{v}=\frac{1}{4}\sum_{i=1}^{4}\delta_{v,i}-\frac{1}{4}\sum_{i=1}^{4}\frac{x_{i}}{\gamma_{i}}$$

That is, the Equation (13) is reduced.

## Appendix B

This appendix deduces the relations of image-domain CTs estimated by the propose method and the TCR Method with the amplitude of real CT.

According to Equation (12), we can get:

(A8) $$\begin{array}{c} \langle M_{HH}M_{HV}^{*}\rangle =A^{2}(<S_{HH}S_{HV}^{*}>+\delta_{R}e^{-j\theta_{2}}\langle {\left|S_{HH}\right|}^{2}\rangle +\delta_{R}e^{-j\theta_{1}}<S_{HH}S_{VV}^{*}>\\ +\delta_{R}e^{j\theta_{1}}<S_{VH}S_{HV}^{*}>+\delta_{R}e^{j\theta_{1}}\langle {\left|S_{HV}\right|}^{2}\rangle ) \end{array}$$

According to the loose reciprocity, the low correlation between co-pol channel and cross-pol channel and the zero phase of $\langle S_{HH}S_{VV}^{*}\rangle$ of objects selected by the evaluation method of isolation, we obtain the energy of $\langle M_{HH}M_{HV}^{*}\rangle$, as follows:

(A9) $$\begin{array}{cc} {\left|\langle M_{HH}M_{HV}^{*}\rangle \right|}^{2}= & A^{4}\{\delta_{R}^{2}{\left[\langle {\left|S_{HH}\right|}^{2}\rangle +X+2\langle {\left|S_{HV}\right|}^{2}\rangle \right]}^{2}\\ & +\delta_{R}^{2}\left\{4X\langle {\left|S_{HV}\right|}^{2}\rangle \left[\cos\left(2\theta_{1}\right)-1\right]+4\langle {\left|S_{HH}\right|}^{2}\rangle \langle {\left|S_{HV}\right|}^{2}\rangle \left[\cos\left(\theta_{1}+\theta_{2}\right)-1\right]\right\}\\ & +\delta_{R}^{2}\left\{2X\langle {\left|S_{HH}\right|}^{2}\rangle \left[\cos\left(\theta_{1}-\theta_{2}\right)-1\right]\right\}\} \end{array}$$

where $X=\left|<S_{HH}S_{VV}^{*}>\right|$.

By inserting Equations (20) and (A9) into Equation (21), $\delta_{v,1}$ can be expressed as:

(A10) $$\begin{array}{cc} \delta_{v,1} & =\delta_{R}\sqrt{1+\frac{\begin{array}{c} 4X\langle {\left|S_{HV}\right|}^{2}\rangle \left[\cos\left(2\theta_{1}\right)-1\right]+4\langle {\left|S_{HH}\right|}^{2}\rangle \langle {\left|S_{HV}\right|}^{2}\rangle \left[\cos\left(\theta_{1}+\theta_{2}\right)-1\right]\\ +2X\langle {\left|S_{HH}\right|}^{2}\rangle \left[\cos\left(\theta_{1}-\theta_{2}\right)-1\right] \end{array}}{{\left[\langle {\left|S_{HH}\right|}^{2}\rangle +X+2\langle {\left|S_{HV}\right|}^{2}\rangle \right]}^{2}}}\\ & =\delta_{R}\sqrt{1+\Delta_{real}}\\ & =\alpha \delta_{R} \end{array}$$

where the second item under the root in the first line of the right-side terms is set as $\Delta_{real}$, and $\sqrt{1+\Delta_{real}}$ in the second line of the right-side terms is signed as α.

Similarly, the relationship of other $\delta_{v,i}$ with $\delta_{R}$ is the same as that of $\delta_{v,1}$ and $\delta_{R}$. So, the image-domain CT estimated by the propose method is:

(A11) $$\delta_{CDT}\approx 2\alpha \delta_{R}$$

At the same time, based on Equation (12), the image domain CT estimated by the TCR method is the following:

(A12) $$\begin{array}{cc} \delta_{TCR} & =2\delta_{R}\sqrt{\frac{1+\cos\left(\theta_{2}-\theta_{1}\right)}{2}}\\ & =2\beta \delta_{R} \end{array}$$

where $\sqrt{\frac{1+\cos\left(\theta_{2}-\theta_{1}\right)}{2}}$ is set as β.

For the assessment of isolation in Section 2.2, the selected object is non-water natural area such as forest. The amplitude of the cross-pol channel of forest is generally about 6.4 dB below the co-pol channel at C band [28], and analysis of RadarSat-2 forest exhibits $\left|\langle {\left|S_{HH}\right|}^{2}\rangle \right|\approx 2X$, so we can get $\Delta_{real}\in \left[-1,0\right]$, then α∈[0,1]. According to Equation (A12), we get β∈[0,1]. In that way, the relationship of $\delta_{CDT}$ with $\delta_{R}$ and that of $\delta_{TCR}$ with $\delta_{R}$ are similar. $\delta_{CDT}$ can correctly reflect the level of $\delta_{R}$, which is similar to the $\delta_{TCR}$ of the TCR method.
